# Functional Roles of Furin in Cardio-Cerebrovascular Diseases

**DOI:** 10.1021/acsptsci.3c00325

**Published:** 2024-02-07

**Authors:** Surasak Wichaiyo, Pimpisid Koonyosying, Noppawan Phumala Morales

**Affiliations:** † Department of Pharmacology, Faculty of Pharmacy, Mahidol University, Bangkok 10400, Thailand; ‡ Centre of Biopharmaceutical Science for Healthy Ageing, Faculty of Pharmacy, Mahidol University, Bangkok 10400, Thailand; § Department of Biochemistry, Faculty of Medicine, Chiang Mai University, Chiang Mai 50200, Thailand; ∥ Department of Pharmacology, Faculty of Science, Mahidol University, Bangkok 10400, Thailand

**Keywords:** Furin, Proprotein convertase subtilisin/kexin type 3, Protein modification, Cardiovascular diseases

## Abstract

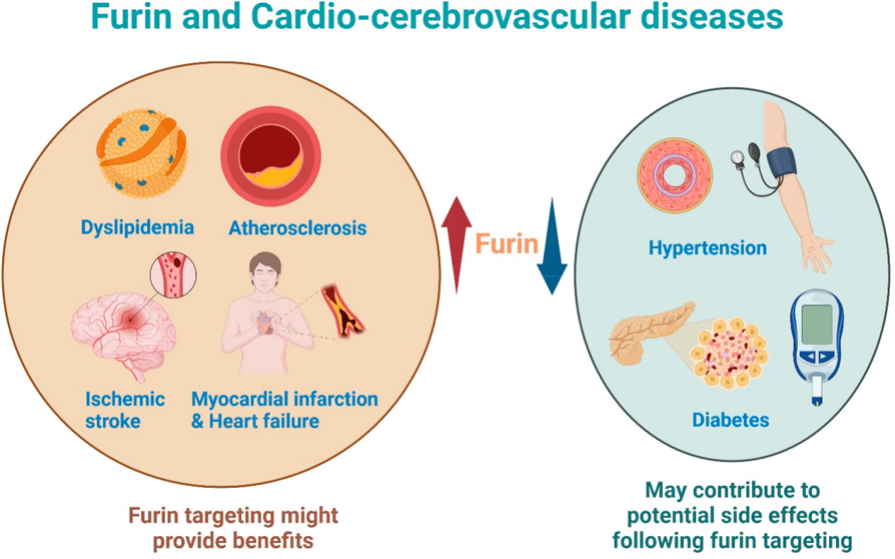

Furin plays a major role in post-translational modification of several biomolecules, including endogenous hormones, growth factors, and cytokines. Recent reports have demonstrated the association of furin and cardio-cerebrovascular diseases (CVDs) in humans. This review describes the possible pathogenic contribution of furin and its substrates in CVDs. Early-stage hypertension and diabetes mellitus show a negative correlation with furin. A reduction in furin might promote hypertension by decreasing maturation of B-type natriuretic peptide (BNP) or by decreasing shedding of membrane (pro)renin receptor (PRR), which facilitates activation of the renin–angiotensin–aldosterone system (RAAS). In diabetes, furin downregulation potentially leads to insulin resistance by reducing maturation of the insulin receptor. In contrast, the progression of other CVDs is associated with an increase in furin, including dyslipidemia, atherosclerosis, ischemic stroke, myocardial infarction (MI), and heart failure. Upregulation of furin might promote maturation of membrane type 1-matrix metalloproteinase (MT1-MMP), which cleaves low-density lipoprotein receptor (LDLR), contributing to dyslipidemia. In atherosclerosis, elevated levels of furin possibly enhance maturation of several substrates related to inflammation, cell proliferation, and extracellular matrix (ECM) deposition and degradation. Neuronal cell death following ischemic stroke has also been shown to involve furin substrates (e.g., MT1-MMP, hepcidin, and hemojuvelin). Moreover, furin and its substrates, including tumor necrosis factor-α (TNF-α), endothelin-1 (ET-1), and transforming growth factor-β1 (TGF-β1), are capable of mediating inflammation, hypertrophy, and fibrosis in MI and heart failure. Taken together, this evidence provides functional significance of furin in CVDs and might suggest a potential novel therapeutic modality for the management of CVDs.

Cardio-cerebrovascular diseases (CVDs) are the leading cause of death globally.^1,2^ A recent report demonstrated that ischemic heart disease and ischemic stroke ranked as the first and second most common causes of cardiovascular (CV) death, respectively.^1^ In addition, hypertension, dyslipidemia, and diabetes mellitus are important modifiable risk factors for CVDs.^1,2^ Current management guidelines for these diseases recommend risk assessment prior to prescribing medications, such as antihypertensive, hypoglycemic, and lipid-lowering agents, together with lifestyle interventions that might help prevent incident CVDs.^3−7^ However, there are several limitations of pharmacological approaches, such as adverse drug reactions^8^ and response variability.^9,10^ Therefore, identification of novel targets that play a major role in the pathogenesis of CVDs might allow the discovery and development of more effective and safer drugs for the management of CVDs.

Furin plays a major role in post-translational modification of several biomolecules, including endogenous hormones, growth factors, and cytokines.^11,12^ In addition, furin has been proposed to promote various pathological contexts, including infections, cancers, and neuropsychiatric disorders.^13−15^ At present, evidence suggests the association between furin expression and CVD risk. Therefore, it would be interesting to understand the functional role of furin in CVDs. This review comprehensively describes the significance of furin in cardiac development and the potential mechanisms of how furin and its substrates might contribute to the pathogenesis of CVDs. These data suggest that furin is a feasible target for the development of novel therapeutic agents in the management of CVDs.

## Expression and Localization of Furin

**Figure 1:**
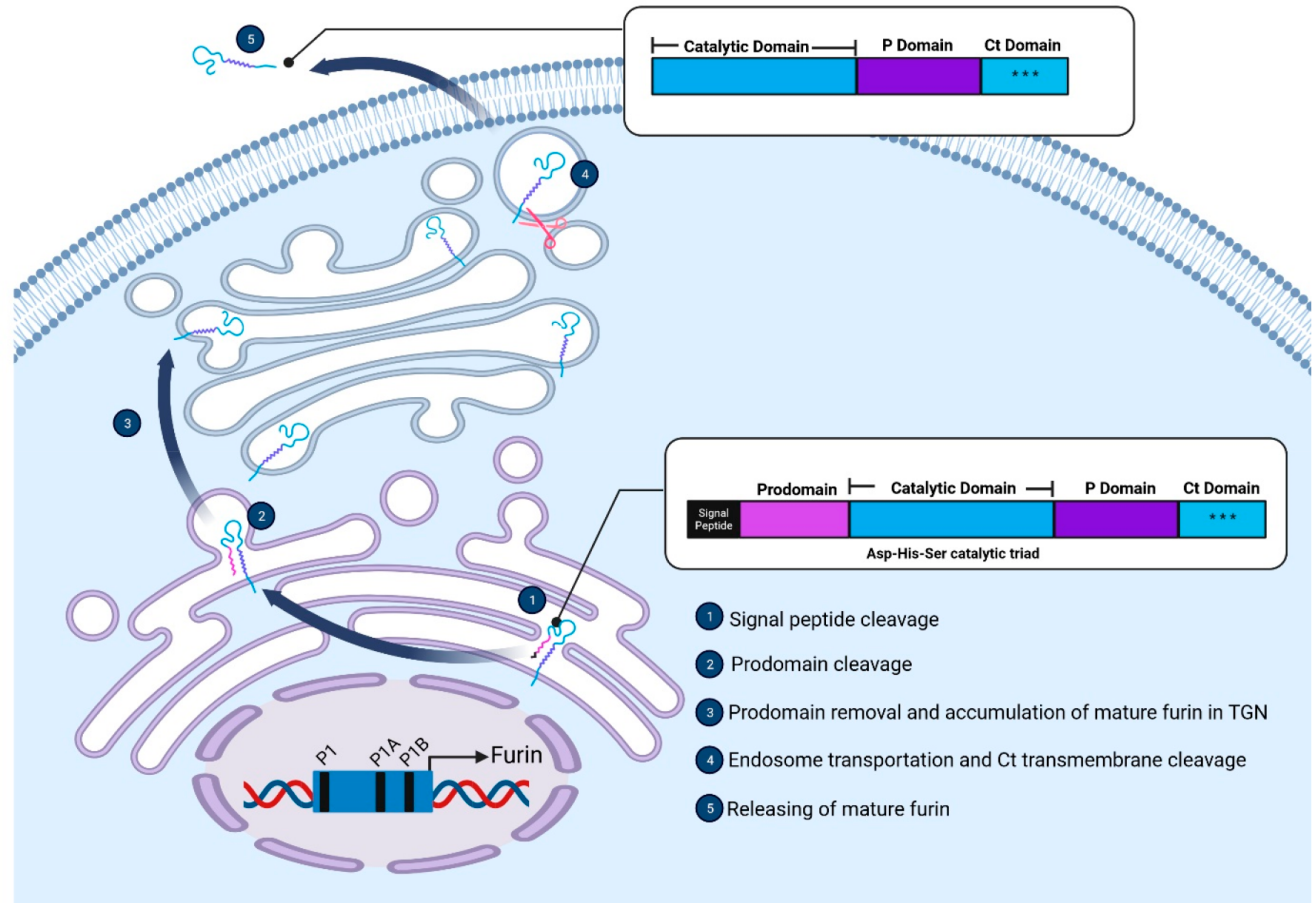
**Furin structure and its localization.** Furin is initially expressed as a proprotein (inactive form). The Ct domain (blue) is inserted into the endoplasmic reticulum (ER) as a transmembrane region. The N-terminal signal peptide (black) is removed via autocatalytic cleavage, which reveals the prodomain region (magenta). Then, the prodomain is cleaved via an autoproteolytic process during trafficking from the ER to the trans-Golgi network (TGN). At this stage, the enzymatic activity of furin is increased due to the exposed catalytic domain (blue). Intracellular mature furin is active and promotes maturation of proproteins in the TGN. Moreover, mature active furin is transported from the TGN to the cell membrane. Proteolytic cleavage of the Ct domain (transmembrane region) allows shedding of mature furin into the extracellular space. (Created with BioRender.com)

Furin is a member of eukaryotic proprotein convertase (PC) family. It is also called proprotein convertase subtilisin/kexin type 3 (PCSK3), given that the PC family has structural similarities to bacterial subtilisin and yeast kexin proteases.^13^ Furin is expressed by the FES upstream region (*FUR*) gene on chromosome 15 of humans,^16,17^ which is regulated by three promoters: P1, P1A, and P1B (Figure 1).^18^ P1 shares the characteristics of cytokine-activated genes, but P1A and P1B are housekeeping genes.^19^ The transcription factor CCAAT/enhancer binding protein (C/EBP) β can bind to the promoters and cooperate with cytokines, such as interferon-γ (IFN-γ), transforming growth factor-β (TGF-β), and interleukin (IL)-12, to stimulate furin transcription. Furin is expressed in early development and is involved in the processing of many proteins.^20,21^ The messenger RNA (mRNA) and protein levels vary depending on the cell type and tissue. High levels of furin are detected in liver, bone marrow, and salivary glands, whereas low levels of furin are observed in muscle cells.

Following translation, furin is initially presented as a proprotein, which is inactive. The Ct domain of furin proprotein is integrated into the endoplasmic reticulum (ER) and acts as a transmembrane region (Figure 1). Post-translational glycosylation of furin also occurs in the ER. Next, the N-terminal signal peptide is removed via autocatalytic cleavage, which reveals a prodomain region.^13,22^ At this stage, the catalytic domain is folded correctly.^19^ Then, the prodomain region is cleaved via an autoproteolytic process during trafficking to the trans-Golgi network (TGN), which increases the enzymatic activity of furin due to the exposed catalytic domain.^23^ Intracellular mature furin is active and promotes maturation of proproteins in the TGN. Moreover, mature or active furin is transported to the cell membrane via endosomes. Proteolytic cleavage of the transmembrane region (the Ct domain) results in shedding of mature furin into the extracellular space. The protease catalytic triad (Asp, His, and Ser) in the catalytic domain plays a role in endoproteolytic processing of many proproteins, including prohormones, growth factors, receptors, membrane channels, adhesion molecules, collagens, metalloproteinases, coagulation factors, and albumin, among others.^24^

## Furin in Cardiac Development

In developmental biology, furin plays an important function in cardiac development. A lack of furin in various cardiac cell types induces cardiac abnormalities in mice. First, homozygous global furin deletion induces lethality at embryonic day 10–11 due to failure of heart morphogenesis and hemodynamic insufficiency.^25^ Second, embryonic lethality is observed in cardiac progenitor cell-specific furin deficiency, in association with decreased proliferation and premature differentiation of cardiac progenitor cells, cardiac abnormalities in the outflow tract, and decreased expression in mature bone morphogenetic protein 4 (BMP4),^26^ which plays a role in embryogenesis, including cardiac development.^27^ Third, furin deficiency in endothelial cells (ECs) leads to ventricular septal defects and/or valve malformations in embryos, which is associated with decreased levels of mature BMP4 and endothelin-1 (ET-1).^28^ Newborns from the EC-specific furin knockout colony die shortly after birth.^28^ Fourth, in the model of furin deletion in differentiated cardiomyocytes, although mice are viable, there is an elongated PR interval,^26^ indicating abnormal cardiac function. Together, these data support the functional role of furin during embryogenesis, at least via post-translational regulation of BMP4 and ET-1, the key players in CV development.^27,29^

## Pathological Contributions of Furin in Cardio-Cerebrovascular Diseases

In CV medicine, furin and its substrates (Table 1) have been demonstrated to play important physiological and pathological roles, including in hypertension, diabetes mellitus, dyslipidemia, atherosclerosis, atherosclerotic cardiovascular diseases (ASCVDs) mainly ischemic stroke and myocardial infarction (MI), and heart failure.

**Table 1:** Substrates of Furin and Their Cardiovascular-Associated Function^a^

Substrate	Product	Function
Pro-BMP4	BMP4	Cardiac development
Pro-BNP	BNP	Vasodilation, diuresis, natriuresis, inhibition of cardiac remodeling, and fibrosis
Pro-ET-1	ET-1	Cardiac development, vasoconstriction, cardiac remodeling, and fibrosis
PRR	Soluble PRR	Regulation of blood pressure and sodium–water homeostasis
ENaC	Active ENaC	Regulation of blood pressure and sodium-water homeostasis
Pro-insulin	Insulin	Glucose and metabolic regulation
Pro-insulin receptor	Insulin receptor	Glucose and metabolic regulation
LPL	Less active LPL	Lipoprotein regulation
Pro-MT1-MMP	MT1-MMP	LDLR shedding, MMP2 maturation and extracellular matrix degradation, cardiac remodeling, and fibrosis
TGF-β1 precursor	Mature TGF-β1	VSMC differentiation, promotion of furin expression on EC, fibroblast differentiation, cardiac remodeling, and fibrosis
Pro-β-NGF	β-NGF	VSMC survival and migration
TNF-α precursor	TNF-α	Inflammation
Notch receptor precursor	Mature Notch receptor	Inflammation
ADAM10 and ADAM17	Active ADAMs	Inflammation (via activation of Notch signaling)
Pro-hepcidin	Hepcidin	Regulation of systemic/cellular iron homeostasis

a Abbreviations: BMP4 = bone morphogenetic protein-4, BNP = B-type natriuretic peptide, ET-1 = endothelin-1, PRR = (pro)renin receptor, ENaC = epithelial sodium channel, LPL = lipoprotein lipase, MT1-MMP = membrane type 1-matrix metalloproteinase, LDLR = low-density lipoprotein receptor, MMP2 = matrix metalloproteinase 2, TGF-β1 = transforming growth factor-β1, VSMC = vascular smooth muscle cell, EC = endothelial cell, β-NGF = β-nerve growth factor, TNF-α = tumor necrosis factor-α, ADAM = a disintegrin and metalloproteinase domain-containing protein.

## Hypertension

The prevalence of hypertension increases with age.^30^ This non-communicable disease cannot be cured by current medical therapy.^31^ In humans, many studies have demonstrated the association of decreased furin and hypertension. Lower serum levels of furin are associated with high blood pressure and might help predict an increased risk of hypertension.^32^ In addition, epigenetic modification by DNA methylation in the promoter region of the *FURIN* gene (suppressed furin expression) is associated with the risk of hypertension (Table 2).^33,34^ Moreover, an individual who carries the 1970C > G (rs2071410) or 5604C > G single nucleotide polymorphism (SNP) in the *FURIN* gene has a significantly lower urinary sodium excretion rate, suggesting decreased furin activity (Table 2),^35,36^ given that furin substrates are responsible for the regulation of sodium excretion. These G alleles are associated with the risk of hypertension.^35,37^ Consistently, a genome-wide association study (GWAS) has reported that the rs4702 A variant of the *FURIN* gene, which results in decreased furin expression, is associated with an increase in systolic blood pressure, diastolic blood pressure, and peripheral vascular resistance.^38^

**Table 2:** Evidence Supporting the Roles of Furin in Cardio-Cerebrovascular Diseases^a^

Study models	Furin expression/activity	Effects	Refs
**Human**			
Epigenetic modification by DNA methylation in the promoter region of the *FURIN* gene	- Suppresses furin expression	- Increases the risk of hypertension	(33, 34)
	- Decreases serum furin	- Increases the risk of diabetes mellitus	(66)
*FURIN* gene	Decreases furin activity	- Increases the risk of hypertension	(35, 36)
1970C > G (rs2071410) or 5604C > G SNP		- Increases the risk of transient ischemic attack	(95)
rs4702 A SNP	Decreases furin expression	- Increases the risk of hypertension	(38)
SNP on 15q26.1 (rs17514846)	Furin upregulation	- Associated with hypertriglyceridemia	(75, 76)
		- Correlated with CAD risk	(96,97)
Clinical study	- High baseline plasma furin	- Increases the risk of diabetes mellitus and mortality	(73)
	- Increased plasma furin after acute MI	- Associated with risk of recurrent MI or cardiovascular events	(98, 99)

**In Vivo**			
β cell-specific furin knockout in mice	Decreases furin activity	β cell dysfunction and glucose intolerance	(67)
Mice treated with homocysteine	Hinders furin cleavage site on pro-insulin receptor	Insulin resistance	(72)
Balloon-induced aortic injury in rats	Furin upregulation	VSMC proliferation	(100)
*Ldlr*^–/–^ and *Apoe*^–/–^ mice treated with a furin inhibitor (α-1-PDX)	Irreversible inhibition of furin	Reduces the size of atherosclerotic lesion and plaque progression	(101)
*Apoe*^–/–^ mice with furin overexpression	Furin upregulation	Promotes plaque formation	(101)
Spontaneous hypertensive rats with MCAO and reperfusion	Furin upregulation	Contributes to neuronal cell death during MCAO	(102)
Rat model of decompensated heart failure	Furin upregulation	Progression of heart failure	(103)

**In Vitro**			
β cells deficient in furin	Decreases furin activity	Reduces cell proliferation and function	(67)
*FURIN* knockdown in EC	Decreases furin activity	- Reduces monocyte migration	(97)
		- Decreases ET-1, VCAM-1, MCP-1, and NF-κB expression	
EC treated with a furin inhibitor (decanoyl-RVKR-CMK)	Decreases furin activity	Reduces inflammatory gene expression	(101)
Macrophages treated with a furin inhibitor (decanoyl-RVKR-CMK)	Decreases furin activity	Reduces monocyte migration and inflammatory gene expression	(101)

a Abbreviations: SNP = single nucleotide polymorphism, CAD = coronary artery disease, MI = myocardial infarction, VSMC = vascular smooth muscle cell, Ldlr^–/–^ = low-density lipoprotein receptor knockout, Apoe^–/–^ = apolipoprotein E knockout, MCAO = middle cerebral artery occlusion, EC = endothelial cell, ET-1 = endothelin-1, VCAM-1 = vascular cell adhesion molecule 1, MCP-1 = monocyte chemotactic protein 1, NF-κB = nuclear factor-kappa B.

**Figure 2:**
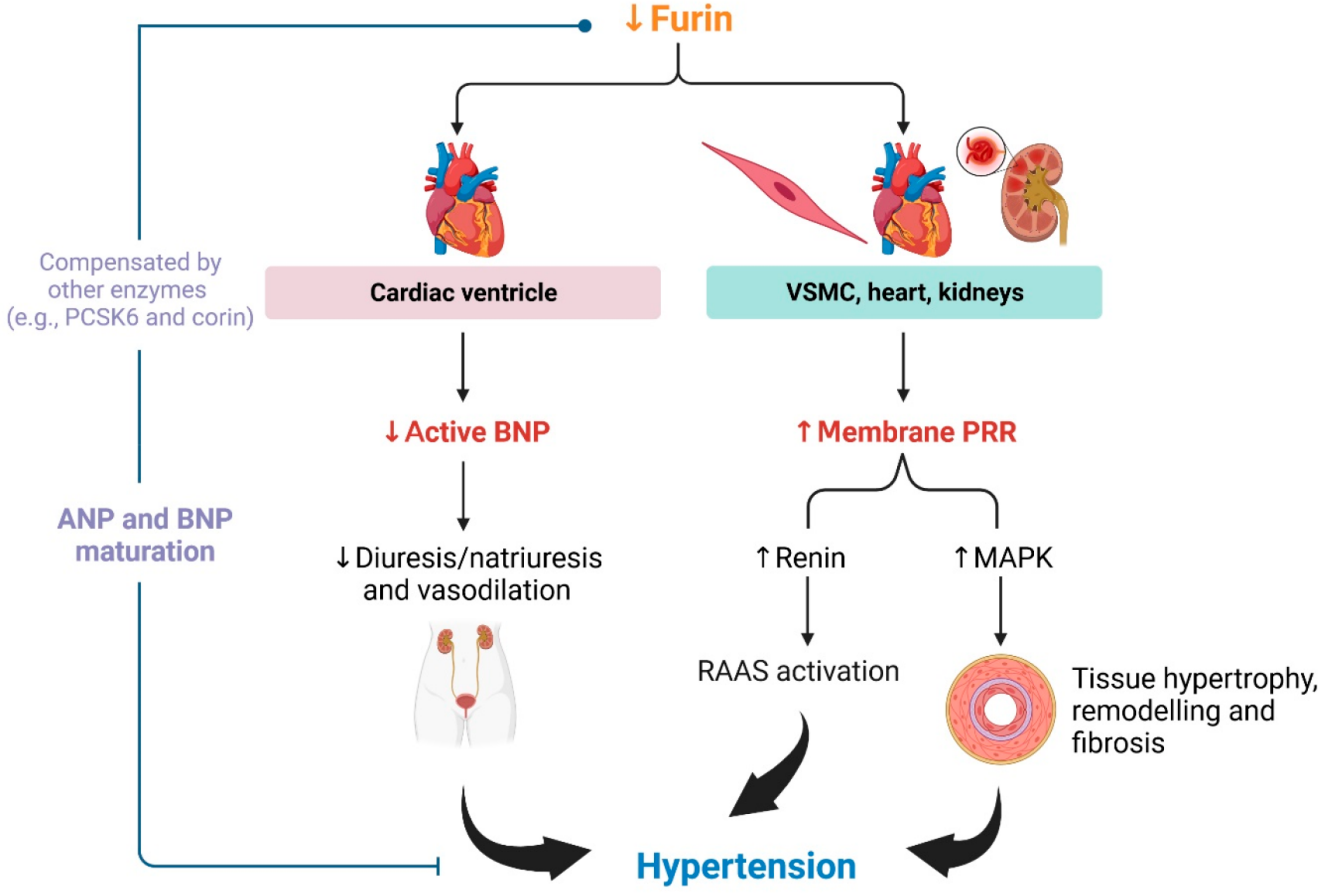
**Possible role of furin in hypertension.** A decrease in furin levels might result in lower levels of mature (active) B-type natriuretic peptide (BNP) and a subsequent reduction in diuresis, natriuresis, and vasodilation, which promotes hypertension, particularly at the early stage. Other enzymes, such as proprotein convertase subtilisin/kexin type 6 (PCSK6) and corin, may compensate for BNP and atrial natriuretic peptide (ANP) maturation in controlling blood pressure. In addition, cleavage of membrane (pro)renin receptors (PRR) might be reduced if furin is downregulated. Membrane PRR converts prorenin to active renin, which activates the renin-angiotensin-aldosterone system (RAAS). Moreover, stimulation of membrane PRR promotes mitogen-activated protein kinase (MAPK) signaling, leading to vascular tissue hypertrophy, remodeling, and fibrosis. Therefore, RAAS and MAPK activations due to furin reduction potentially contribute to hypertension. (Created with BioRender.com)

Alterations in B-type natriuretic peptide (BNP) and (pro)renin receptor (PRR) might contribute to hypertension following furin reduction (Figure 2). Furin plays a role in cleaving pro-BNP to active BNP before it is secreted from cardiac ventricles.^39,40^ BNP promotes vasodilation, diuresis, and natriuresis, but inhibits cardiac remodeling and fibrosis.^40−42^ These activities compensate for the cardiac function in response to volume overload, myocardial stretch, elevated angiotensin II (ATII) levels, increased sympathetic outflow, and vasoconstriction.^40−42^ The blood-pressure-lowering effect of BNP against ATII has been demonstrated in dogs.^43^ Therefore, a reduction in furin may decrease mature BNP levels, thus leading to hypertension (Figure 2). Consistently, BNP knockout Dahl salt-sensitive rats present hypertension, in association with left ventricular hypertrophy,^44^ and abnormal function in the heart (i.e., cardiac stiffness, fibrosis, QT interval prolongation, and thrombosis) and the kidneys (i.e., glomerular damage, proteinuria, and fibrosis).^44^ The human data revealed that deficiency in BNP activation is observed in the early stage, including pre-hypertension and stage 1 hypertension,^45,46^ supporting the reports of furin reduction in hypertension. However, BNP levels are increased in the later stages of hypertension,^45^ suggesting the alteration of furin expression and/or the compensatory role of other enzymes, such as PCSK6 and corin.^47^ These enzymes also play an essential role in blood pressure control via the activation of atrial natriuretic peptide (ANP).^48−50^

PRR is a receptor for prorenin and renin, which is widely expressed (e.g., in the brain, heart, kidneys, adrenal gland, and pancreas).^51−54^ Binding of prorenin/renin with membrane-bound PRR promotes hypertension, inflammation, tissue remodeling, and fibrosis^51−54^ by activating prorenin (to active renin) and subsequent AT II generation, or by stimulating mitogen-activated protein kinases (MAPKs), including extracellular signal-regulated kinase 1/2 (ERK1/2) signaling, which leads to upregulation of cyclooxygenase-2, TGF-β1, collagen, and fibronectin.^51−54^ Soluble PRR is generated by furin, and it may localize inside the cells or be secreted.^51−54^ Therefore, a reduction in furin levels may retain the intact membrane PRR to mediate pathological processes (Figure 2). Notably, the current evidence suggests that soluble PRR also produces pathobiological functions, including sodium–water retention (via activation of epithelial sodium channel [ENaC]), hypertension (via the local renin–angiotensin–aldosterone system [RAAS]), and heart failure.^53,55^ However, there are inconsistencies between preclinical and human data. Several animal studies have demonstrated the association between soluble PRR and hypertension.^56−60^ In patients with essential hypertension, serum soluble PRR levels are not associated with blood pressure; rather, soluble PRR levels appear to reflect reduced renal function.^61^

ENaC also requires activation by furin before it participates in regulating sodium re-absorption and vascular tone.^62^ A recent systematic review of human studies reported that pharmacological inhibition of ENaC have no statistically or clinically significant blood-pressure-lowering effect,^63^ indicating that decreased ENaC activity due to furin reduction might not have a significant impact on blood pressure.

## Diabetes Mellitus

Diabetes mellitus is characterized by hyperglycemia due to a reduction in insulin secretion and/or insulin resistance.^64^ In patients with diabetes, serum furin is decreased, relative to individuals with normal plasma glucose levels,^65^ indicating the association between lower serum furin levels and the risk of pre-diabetes and diabetes.^65^ Similarly to what is seen in hypertension, a study with 4 years of follow up found that non-diabetic individuals with DNA hypermethylation of the promoter region of the *FURIN* gene might have an increased risk of diabetes (Table 2).^66^

**Figure 3:**
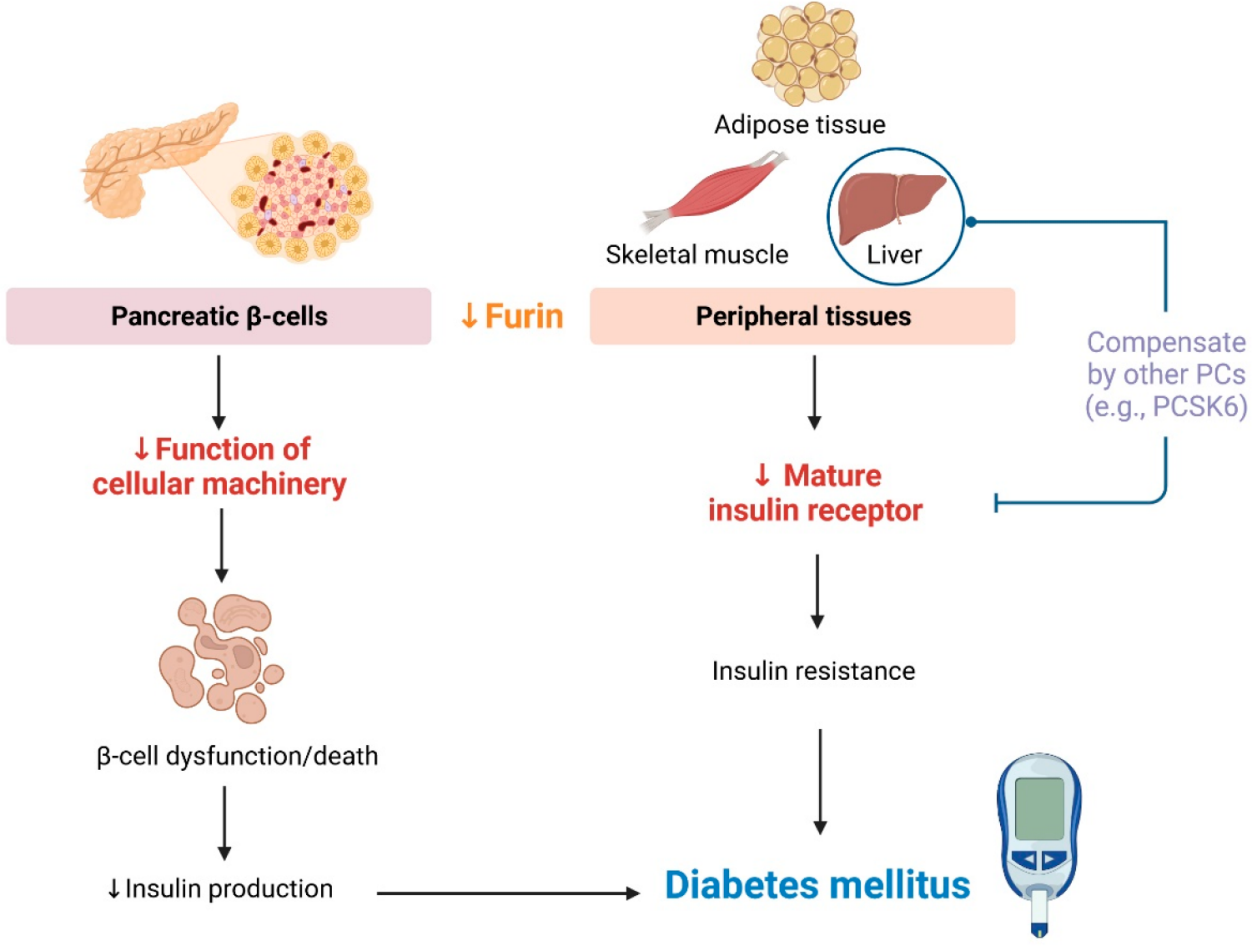
**Proposed mechanisms for the role of furin in promoting diabetes mellitus.** A reduction in furin during diabetes contributes to decreased function of cellular machinery in pancreatic β cells, resulting in β cell dysfunction and death. In peripheral tissues, including skeletal muscle, adipose tissue, and liver, furin downregulation might lead to insulin resistance by decreasing the maturation of insulin receptors. However, there is less pronounced impairment of insulin receptor maturation in the liver, possibly due to a compensatory function from other proprotein convertases (PCs), including proprotein convertase subtilisin/kexin type 6 (PCSK6). (Created with BioRender.com)

Pancreatic β cell dysfunction and insulin resistance might occur if furin is downregulated. Furin is highly expressed in pancreatic islets. Mice with β cell–specific furin knockout are glucose intolerant^67,68^ and have lower plasma insulin levels, in association with β cell dysfunction, denoted by smaller islets, decreased β cell density, and lower insulin content (Table 2).^67^ Although furin is capable of mediating the maturation of insulin,^69^ there are no defects in insulin maturation in mice with β cell–specific furin knockout,^67^ suggesting the compensatory function of other PCs, such as PC1 and PC2.^70^ An *in vitro* study in murine pancreatic β cells suggested that furin deficiency attenuates cell proliferation (Table 2),^67^ given that furin regulates intracellular machinery that is crucial for β cell growth and survival, including the ATPase proton pump, protein cargo (e.g., secretory granules and lysosomes), enzymes, transporters, and stress-related genes (Figure 3).^67^ Moreover, the pro-insulin receptor is a furin substrate.^68,71^ Data from mice have shown that using homocysteine to hinder furin binding to the pro-insulin receptor prevents cleavage of the pro-insulin receptor in peripheral tissues, including muscle, adipose tissue, and, to a lesser extent, the liver; this impaired cleavage promotes insulin resistance (Table 2).^72^ The less pronounced impairment of pro-insulin receptor cleavage in the liver is supported by observations in mice with liver-specific furin knockout, suggesting a redundant function of other PCs for insulin receptor maturation in this organ.^68^ Taken together, this evidence supports the association between furin reduction and diabetes (Figure 3).

In an opposite manner, a study with 21 years of follow up demonstrated that baseline plasma furin levels positively correlate with increased risk of diabetes and mortality in humans (Table 2), indicating the role of furin in diabetes development.^73^ Although this study suggested that individuals might have elevated plasma furin levels for several years before the onset of diabetes, a potential limitation is measuring furin levels just once at baseline.^73^ To fully understand this observation, future studies elucidating the fluctuation of furin expression during diabetes progression, together with alterations of other furin substrates, are needed.

## Dyslipidemia

**Figure 4:**
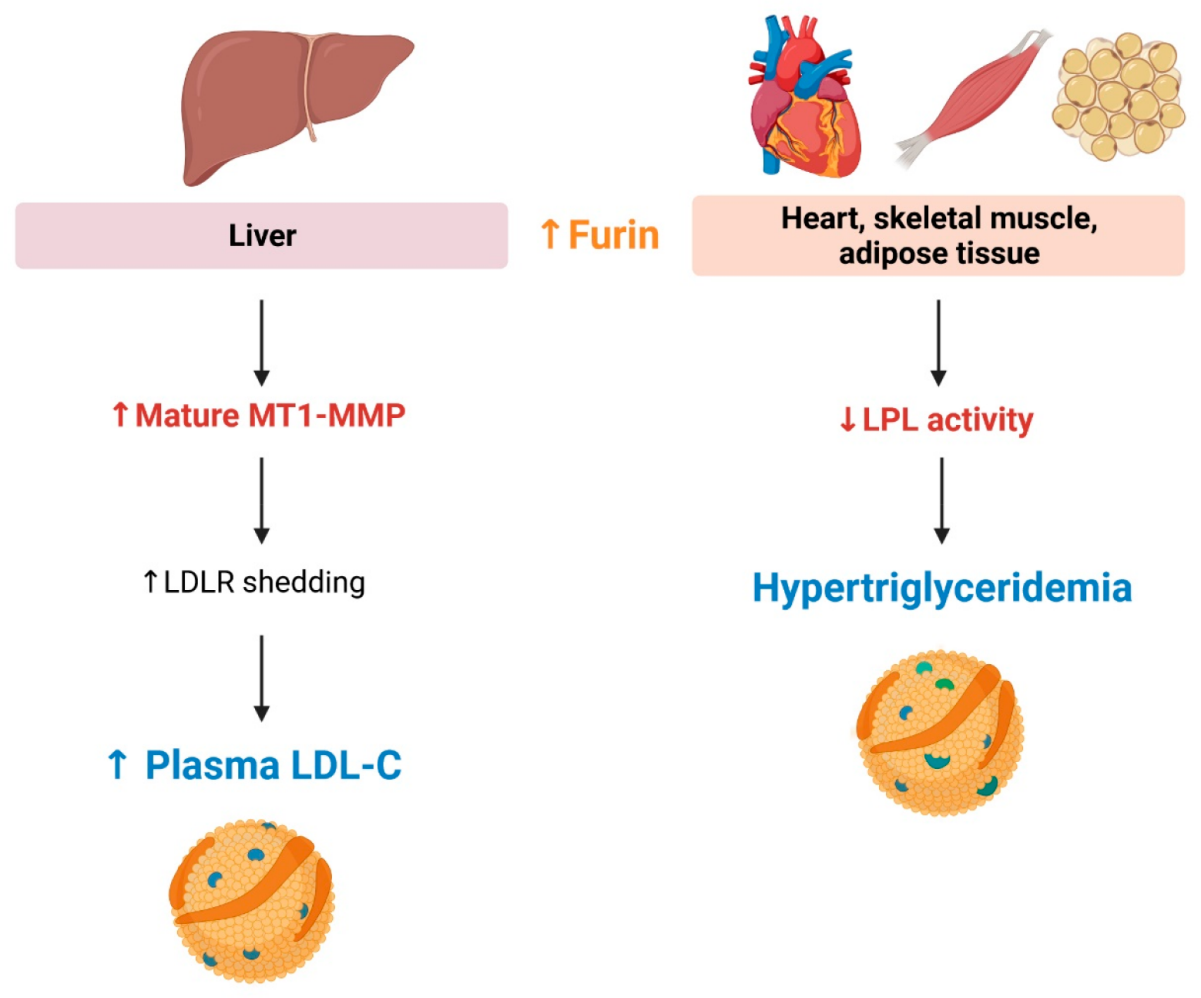
**Potential contribution of furin in dyslipidemia.** Increased furin levels may contribute to hypertriglyceridemia, given that furin-cleaved lipoprotein lipase (LPL) is less active in triglyceride catabolism. In addition, the elevated furin levels may activate maturation of membrane type 1-matrix metalloproteinase (MT1-MMP), which mediate the shedding of low-density lipoprotein receptor (LDLR), leading to increased plasma levels of low-density lipoprotein cholesterol (LDL-C). (Created with BioRender.com)

Dyslipidemia is one of the most common chronic conditions throughout the world.^74^ Meta-analyses of GWAS in Caucasian populations have revealed that a SNP on chromosome 15q26.1 (rs17514846), which is located in the *FURIN* gene, results in furin upregulation, and it is associated with dyslipidemia, particularly hypertriglyceridemia (Table 2).^75,76^ Triglyceride lipase family members, including lipoprotein lipase (LPL) and endothelial lipase (EL), are furin substrates.^77,78^ Furin-cleaved LPL and EL are less active in triglyceride catabolism.^77,78^ However, hypertriglyceridemia following increased furin levels might be due to LPL malfunction, given that EL primarily hydrolyzes phospholipids in high-density lipoprotein cholesterol (HDL-C), contributing to a reduction in HDL-C particle size.^79−83^ LPL is abundantly synthesized by the heart, skeletal muscle, and adipose tissue, then released and anchored at luminal sites of ECs.^84^ Researchers have demonstrated a significant role of LPL in triglyceride hydrolysis based on the observation of severe hypertriglyceridemia in homozygous global LPL knockout in mice, which leads to postnatal death.^84,85^ Heterozygous LPL-deficient mice survive but display hypertriglyceridemia.^84^ Similarly, adult mice (8 weeks old) with cardiac-specific deletion of LPL have increased plasma triglyceride levels and cardiac dysfunction (Figure 4).^86^

Furin upregulation might also affect low-density lipoprotein cholesterol (LDL-C) levels. There is abundant evidence that LDL-C is a strong etiological factor of atherosclerosis and adverse CV events.^87−89^ LDL-C is taken up by hepatocytes; this uptake is a major mechanism for lowering plasma LDL-C levels. Following binding of LDL-C to the low-density lipoprotein receptor (LDLR) on the surface of hepatocytes, this ligand–receptor complex is internalized to hydrolyze LDL-C.^87^ Recently, membrane-type 1 matrix metalloproteinase (MT1-MMP) has been demonstrated to play a role in LDLR shedding. MT1-MMP colocalizes with the LDLR in hepatocytes *in vitro*.^90^ In addition, mice with liver-specific MT1-MMP deletion have increased LDLR in the liver, but lower plasma levels of soluble LDLR, in association with a reduction in plasma cholesterol levels.^90^ Given that furin is responsible for the MT1-MMP maturation,^91^ an increase in furin activity might elevate plasma LDL-C levels by promoting LDLR shedding (Figure 4).^90^ Although PCSK9 is another furin substrate,^92,93^ furin-cleaved PCSK9 remains active in LDLR degradation and cholesterol catabolism.^94^ Therefore, PCSK9 might not be a primary target of furin in promoting dyslipidemia.

## Atherosclerosis

Atherosclerosis is a chronic inflammatory disease of the arteries due to plaque development.^104^ Interestingly, high furin levels are detected within human atherosclerotic plaques, including in the carotid artery, aorta, and femoral artery.^105^ Consistently, a GWAS revealed that *FURIN* rs17514846 strongly correlates with CAD risk in humans (Table 2).^96^ Macrophages from individuals carrying the A/A genotype for rs17514846 (the CAD risk allele) have higher *FURIN* expression than those carrying the C/C genotype. In addition, ECs collected from individuals carrying the A/A genotype for rs17514846 present furin upregulation, in association with higher circulating levels of monocyte chemotactic protein-1 (MCP-1) and increased thickness of the carotid intima-media.^97^

**Figure 5:**
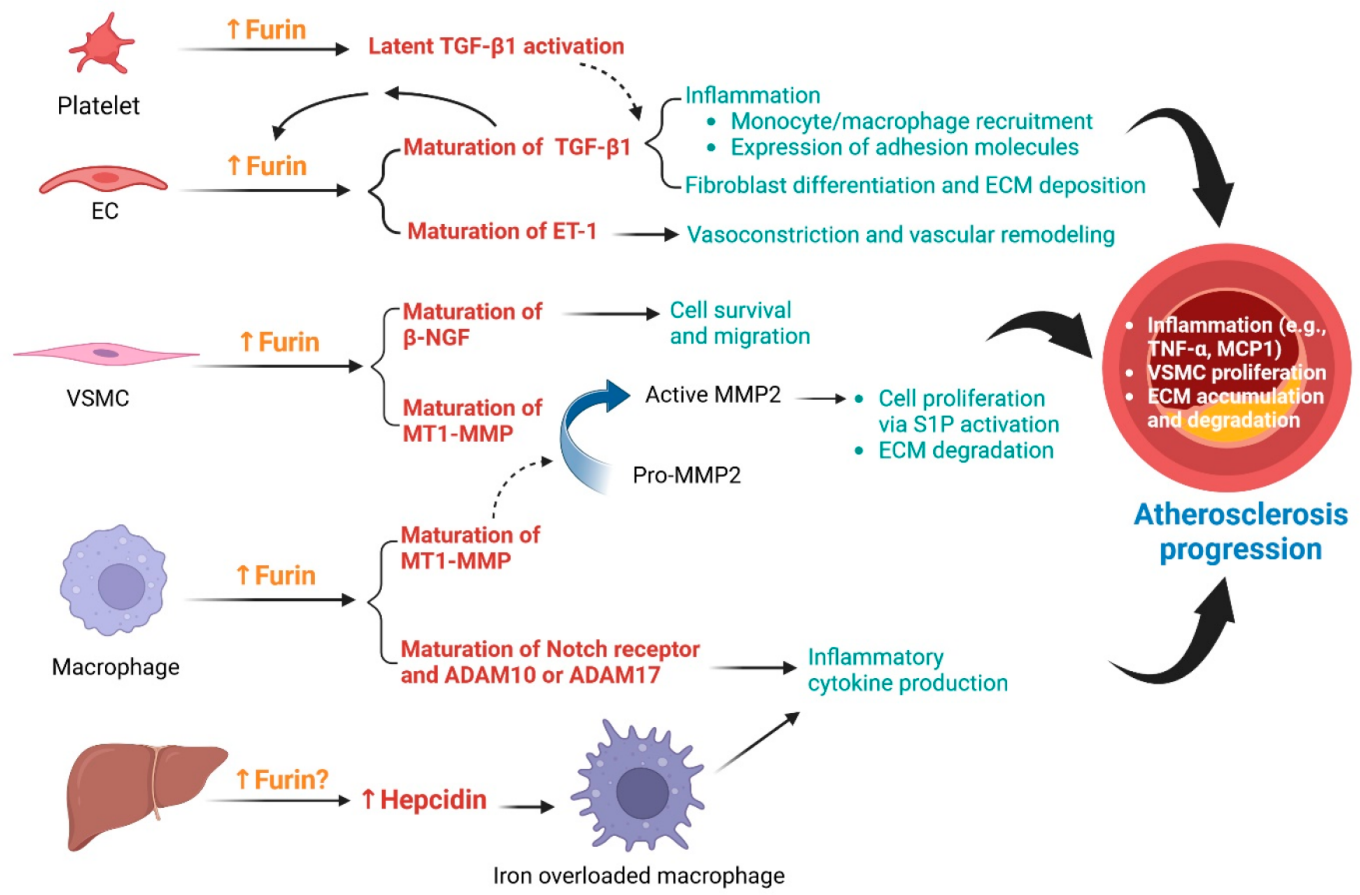
**Function of furin in the progression of atherosclerosis.** Furin expression is elevated during atherosclerotic progression. In vascular endothelial cells (ECs), furin promotes maturation of endothelin-1 (ET-1), which contributes to vasoconstriction and vascular remodeling. Maturation of transforming growth factor-β1 (TGF-β1) in ECs is also mediated by furin. TGF-β1 stimulates inflammation, fibroblast differentiation and extracellular matrix (ECM) deposition. In addition, TGF-β1 is capable of activating furin expression in ECs. Moreover, furin is secreted by activated platelets, which promotes latent TGF-β1 activation. In vascular smooth muscle cells (VSMCs), furin processes active β-nerve growth factor (β-NGF), which promotes survival and migration of the cells. Maturation of membrane type 1-matrix metalloproteinase (MT1-MMP) in VSMCs and macrophages is mediated by furin. MT1-MMP activates matrix metalloproteinase 2 (MMP2), which regulates cell migration (via stimulation of sphingosine-1 phosphate [S1P]) and ECM degradation. In macrophages, furin also processes the maturation of Notch signaling-related molecules, including Notch receptors, A disintegrin and metalloproteinase domain-containing protein 10 (ADAM10) and ADAM17, which promote inflammation. Moreover, a liver-derived furin substrate, hepcidin, is increased and plays a role in limiting macrophage iron export, which results in macrophage iron accumulation and inflammation. (Created with BioRender.com)

Furin is responsible for various pathophysiological processes of atherosclerosis, including lipid metabolism (Figure 4), inflammation, and VSMC proliferation.^11^ Furin is expressed on ECs, VSMCs, and monocytes/macrophages.^101^ An *in vitro* study showed that *FURIN* knockdown in ECs reduces the secretion of ET-1, a furin substrate vasoactive peptide,^106^ as well as the expression of vascular cell adhesion molecule 1 (VCAM-1), MCP-1, and nuclear factor-kappa B (NF-κB).^97^ These alterations are associated with a reduction in monocyte adhesion and trans-endothelial migration (Table 2), suggesting that EC-expressed furin may promote endothelial activation and inflammation (e.g., monocyte/macrophage recruitment) during the progression of atherosclerosis (Figure 5).^97^ Consistently, researchers found that decanoyl-RVKR-CMK, a competitive furin inhibitor, reduces inflammatory gene expression in tumor necrosis factor-α (TNF-α)-stimulated primary human coronary artery ECs, including VCAM-1, MCP-1, NF-κB, and IL-1β (Table 2).^101^ Moreover, shear stress flow promotes upregulation of TGF-β1 and furin on bovine aortic ECs *in vitro*.^107^ TGF-β1 induces furin expression, whereas furin processes TGF-β1 precursor to an active form during shear flow setting.^107^ Arterial specimens taken proximal to the aortic arch following carotid arteriovenous shunt formation in rabbits showed that TGF-β1 and furin are co-expressed on ECs.^107^ Given that mature TGF-β1 promotes inflammation, fibroblast differentiation, and matrix deposition (Figure 5),^108^ endothelial upregulation of TGF-β1 and furin during disturbed shear stress potentially contributes to atherosclerotic plaque formation, plaque vulnerability, stent restenosis, and intimal hyperplasia.^104,109^ In addition to ECs, activated platelets are capable of secreting furin, which promotes latent TGF-β1 activation and potentially mediates the progression of atherosclerosis.^110,111^

Furin promotes survival and migration of VSMCs by mediating the maturation of β-nerve growth factor (β-NGF).^100,112^ VSMCs stimulated with platelet-derived growth factor BB (PDGF-BB), a pro-proliferative cytokine and TGF-β1 (a pro-differentiating cytokine) have increased furin activity and β-NGF levels (Figure 5).^112^ β-NGF promotes *in vitro* migration of VSMCs in a concentration-dependent manner.^112^ Moreover, furin is upregulated in proliferating VSMCs following balloon-induced aortic injury in rats,^100^ suggesting its role in VSMC proliferation (Figure 5). Consistently, furin may convert pro-MT1-MMP to mature MT1-MMP, which further mediates the maturation of matrix metalloproteinase 2 (MMP2) to promote proliferation of TNF-α-stimulated VSMCs through the induction of sphingolipid metabolism (Figure 5).^91^

In monocytes/macrophages, furin contributes to cell proliferation and migration, but protects against cell apoptosis.^96^ A furin inhibitor attenuates *in vitro* monocyte migration and inflammatory cytokine gene expression, including VCAM-1, intercellular adhesion molecule 1 (ICAM-1), MCP-1, and IL-1β in stimulated monocytes/macrophages (Table 2),^101^ suggesting the role of furin in promoting inflammatory macrophages. A recent study suggested that furin might drive inflammation by activating Notch signaling (Figure 5). This phenomenon includes maturation of Notch receptors,^113−115^ A disintegrin and metalloproteinase domain-containing protein 10 (ADAM10),^116^ and ADAM17.^117^ An intracellular precursor of Notch receptors is cleaved by furin before trafficking to the plasma membrane. Upon activation by its ligands, the extracellular domain of Notch receptors is cleaved by ADAMs, and the remaining transmembrane domain is cleaved by γ-secretase, finally releasing the intracellular domain into the cytoplasm.^113−115^ The Notch intracellular domain is capable of translocating to nucleus and promoting the expression of genes, including inflammatory cytokines.^113−115^ An *in vitro* study revealed that lipopolysaccharide (LPS) induces furin upregulation and Notch activation, in association with the production of inflammatory cytokines in macrophages.^118^ In atherosclerosis, Notch signaling promotes the inflammatory phenotype of macrophages.^119^ Moreover, an *in vitro* study revealed that furin mediates the maturation of MT1-MMP in macrophages, which later activates MMP2 secreted from other cells, including VSMCs (Figure 5).^120^

The functional significance of furin has been supported by animal models of atherosclerosis. In male LDLR knockout (*Ldlr*^–/–^) mice fed a Western diet, α-1-PDX (an irreversible furin inhibitor) reduces the size of the atherosclerotic lesion, macrophage infiltration, MMP2 activity, and collagen accumulation in aortic sinus (Table 2).^101^ Given that mature MMP2 is an indirect furin substrate^91^—it plays an important role in extracellular matrix degradation and contributes to a weakened plaque cap^101^—reduced MMP2 activity following treatment with a furin inhibitor might protect against plaque rupture. There was a similar observation in male apolipoprotein E knockout (*Apoe*^–/–^) mice fed a Western diet and subjected to wire-induced endothelial injury of the common carotid artery.^101^ The authors found that the furin inhibitor reduces the thickness of the intima, macrophage infiltration, TNF-α levels, and the number of VSMCs in the plaque area.^101^ In contrast, furin overexpression promotes neointimal plaque formation in *Apoe*^–/–^mice with a wire injury model of atherosclerosis (Table 2),^101^ supporting the role of furin in promoting atherosclerotic plaque progression *in vivo* (Figure 4). However, the role of furin *in vivo* has been complicated by a recent study demonstrating that an increase in furin might suppress the progression of atherosclerosis.^121^ Female *Apoe*^–/–^ mice fed a high-fat diet and with furin overexpression have enhanced macrophage autophagy, attenuated growth of intra-aortic plaques, and reduced plaque vulnerability.^121^ In addition, aortic tissues from five patients who underwent the Bentall procedure showed increased furin levels and autophagic markers in the plaque.^121^ Whether this controversial observation is a result of sex difference requires further research.

Moreover, hepcidin, a central iron regulator, has been shown to promote atherosclerosis. Prohepcidin is cleaved by furin (to active hepcidin) before being secreted by hepatocytes.^122−124^ Hepcidin limits iron absorption by enterocytes and reduces iron recycling by macrophages.^125^ Mice with hepcidin and LDLR knockout (*Hamp*^–/–^, *Ldlr*^–/–^) present a reduction in macrophage iron, an aortic macrophage inflammatory phenotype, and aortic lipid accumulation.^126,127^ Therefore, it has been proposed that increased hepcidin levels (e.g., due to furin upregulation) attenuate iron mobilization from macrophages. The iron accumulation in macrophages promotes atherogenesis, including oxidative stress and inflammation (Figure 5).^126−128^ Consistently, serum hepcidin levels are associated with atherosclerosis in postmenopausal women.^128^

## Ischemic Stroke

**Figure 6:**
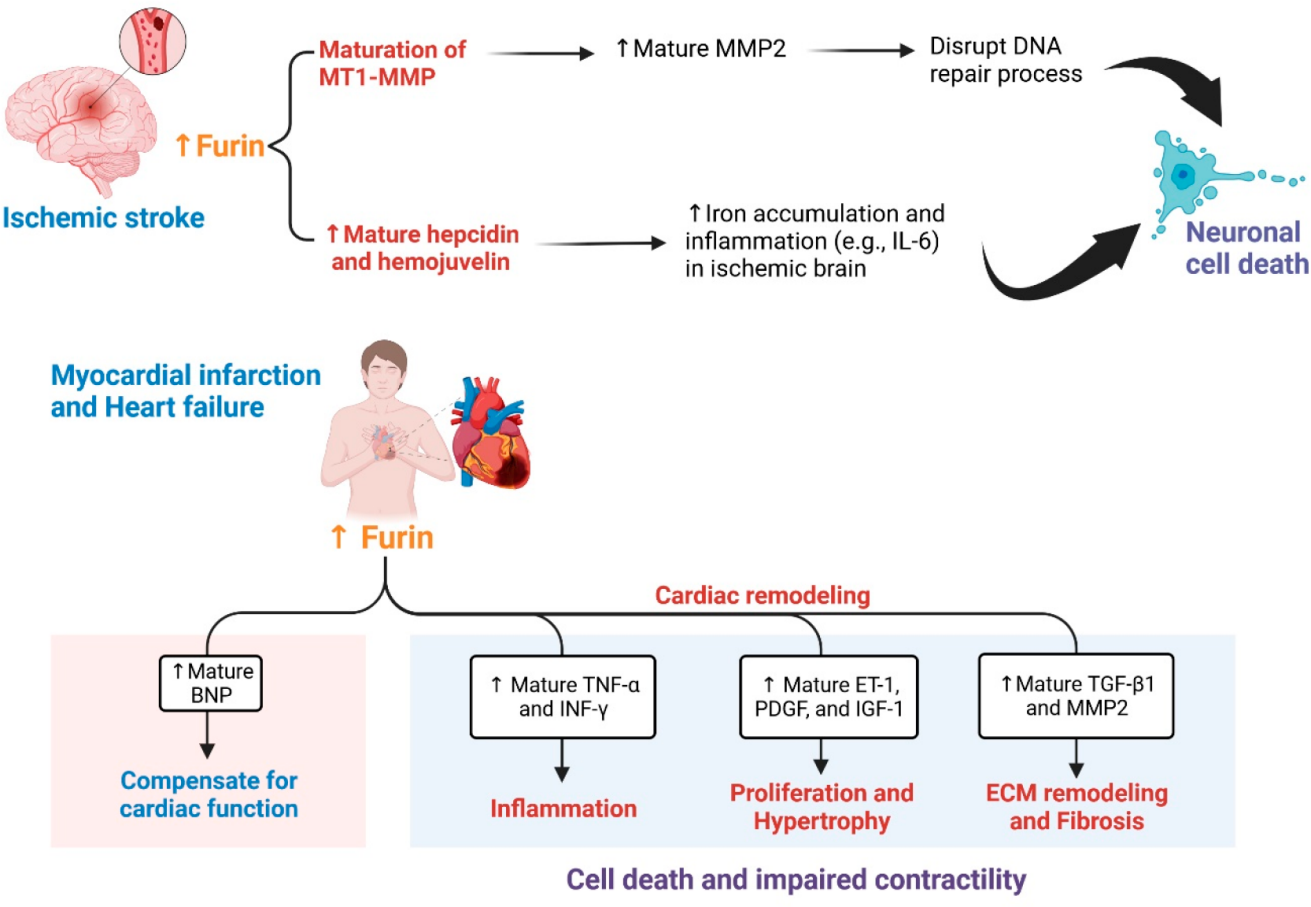
**Mechanisms responsible for the pathogenic role of furin in ischemic stroke, myocardial infarction, and heart failure.** After ischemic stroke, the increase in furin promotes maturation of membrane type 1-matrix metalloproteinase (MT1-MMP), which activates matrix metalloproteinase 2 (MMP2) in ischemic cells. MMP2 attenuates DNA repair due to oxidative damage following ischemic/reperfusion in the brain, leading to neuronal cell death. Hepcidin and hemojuvelin, which regulate iron levels, are also elevated, and thus contribute to iron accumulation and inflammation in the ischemic brain. After acute myocardial infarction and in heart failure, furin may contribute to cardiac remodeling by processing the precursors of several substrates, including (1) inflammatory mediators, such as tumor necrosis factor-α (TNF-α) and interferon-γ (IFN-γ); (2) factors that promote fibrosis, such as transforming growth factor-β1 (TGF-β1) and MMP2; and (3) hypertrophic factors, such as endothelin-1 (ET-1), platelet-derived growth factor (PDGF), and insulin-like growth factor-1 (IGF-1), which result in cell death and impaired contractility. Moreover, elevation of B-type natriuretic peptide (BNP) due to furin upregulation might partially compensate for cardiac function. (Created with BioRender.com)

To emphasize rapid interventions, which help prevent neuronal loss during ischemic stroke, the phrase “time is brain” is widely used.^129^ Nevertheless, the success rate for complete re-canalization following intravenous thrombolytic therapy is less than 50%, suggesting the need for novel therapeutic agents.^130^ A genetic study reported that the CG/GG genotypes of the *FURIN* rs2071410 SNP are associated with ∼50% increased risk of transient ischemic attack (TIA), compared with the homozygous CC genotype (Table 2).^95^ This SNP potentially contributes to a reduction in furin activity and an increase in hypertension,^35^ a strong associated risk factor of TIA.^131^ In stroke, however, GWAS meta-analyses have revealed that *FURIN* is one of the putative causal genes.^132^ This observation is supported by a study in spontaneous hypertensive rats with middle cerebral artery occlusion (MCAO) and reperfusion that demonstrated upregulation and co-localization of furin and MT1-MMP (the activators of MMP2) in ischemic cells (Table 2).^102^ In addition, MMP2 and MMP9 activity in the nucleus is increased in the ischemic hemisphere.^102^ In this model, the authors suggested that ischemia/reperfusion injury induces oxidative DNA damage. The increase in intranuclear MMPs disrupts the DNA repair process, which contributes to cell death (Figure 6).^102^

Another murine MCAO model showed that expression of furin substrates, including hepcidin^122,123^ and hemojuvelin,^133,134^ is increased in the ischemic brain.^135^ Given that hepcidin is upregulated due to iron overload and inflammation,^128^ this pathological process in the brain might contribute to neuronal damage during ischemia-reperfusion. Consistently, the hepcidin level is significantly elevated, in association with IL-6 upregulation and iron accumulation, in the ischemic brain following MCAO in rats (Figure 6).^136^ The elevated serum hepcidin levels, in association with serum iron profiles and IL-6 levels, are also detected in childhood-onset ischemic stroke, which emphasizes the pathogenic contribution of furin and hepcidin in mediating iron overload and inflammation in ischemic stroke.^137^

Moreover, researchers suggest that hemojuvelin might act as a prognostic marker of acute ischemic stroke, given that patients with acute ischemic stroke have increased brain and plasma levels of hemojuvelin.^135^ This protein acts as a dietary iron sensor and hepcidin activator.^138^ Mice with hemojuvelin deficiency present a smaller infarct area and lower levels of apoptotic proteins, including cleaved caspase-3,^135^ evidence that again supports the role of iron overload in ischemic stroke. Notably, the evidence also indicates that furin is involved in the maturation of several thrombogenic factors, including von Willebrand factor,^139,140^ clotting factor VIII,^141,142^ and clotting factor IX.^143^ Additional studies are required to understand the association between furin and these factors in cardio-cerebrovascular thrombosis.

## Myocardial Infarction

In a similar manner to ischemic stroke, the phrase “time is muscle” reflects the need for an early initiation of revascularization following acute MI.^129,144^ Recent evidence indicates that plasma furin levels in patients after acute MI are strongly associated with the risk of recurrent nonfatal MI alone^98^ or recurrent CV events (i.e., a composite of all-cause mortality, hospitalization for heart failure, and recurrent MI) (Table 2).^99^ In addition, furin might act as a better prognostic indicator than BNP and troponin I.^99^ Moreover, bioinformatic analysis revealed that furin may contribute to cardiac remodeling after acute MI by processing the precursors of several substrates, including (1) TNF-α and IFN-γ, which drives inflammation; (2) TGF-β1 and MMP2 for extracellular matrix remodeling and fibrosis; and (3) ET-1, PDGF, and insulin-like growth factor-1 that promote hypertrophy, subsequently leading to cell death and impaired contractility (Figure 6).^145^ Therefore, furin inhibition has been proposed as a potential cardioprotective strategy after MI.^145^

Notably, furin-mediated generation of active BNP is increased in a rat model of MI induced by coronary artery ligation,^146^ supporting the increase in furin and the compensatory role of BNP (Figure 6). In patients with MI, plasma BNP increases up to 60-fold within 24 h after the infarction, and the levels decrease gradually.^147,148^ A second peak might be detected approximately 5 days later, which is reflective of cardiac remodeling.^147,148^ Similarly to an increase in furin, an increase in BNP during MI is associated with all-cause mortality and major adverse cardiovascular events (MACEs), which is a composite endpoint of all death, any MI, and any revascularization.^149,150^

## Heart Failure

Given that heart failure represents the final outcome of various heart diseases,^151^ its pathological mechanisms are multifactorial.^152^ These include inflammation, oxidative stress, mitochondrial dysfunction, abnormal calcium handling, and endothelial dysfunction, which lead to cardiac remodeling, hypertrophy, and fibrosis.^152,153^ In a rat model of decompensated heart failure, the authors noted upregulation of furin in the left ventricle (Table 2), which potentially contributes to the progression of heart failure (Figure 6).^103^ In addition, there is elevated BNP protein and furin in a canine model of early stage heart failure, in association with collagen deposition in left atria and ventricle.^154^

In patients with acute decompensated heart failure and chronic heart failure, furin activity is increased due to myocardial stress, which contributes to the elevated levels of BNP to compensate for cardiac function.^39^ Consistently, several furin substrates have been shown to contribute to the pathogenesis of heart failure (Figure 5), including inflammatory (TNF-α), remodeling/fibrosis (TGF-β1 and MMP2), and hypertrophic (soluble PRR-mediated AT II, BNP, and ET-1) factors, supporting the role of furin in this setting.^53,55,155−157^

## Conclusions

The observations regarding the association between furin and CVDs in humans have captured the attention of biomedical scientists to understand the potential mechanisms of how this proprotein-converting enzyme contributes to CVDs. Based on the available data, furin shows a positive correlation with the progression of most CVDs, including dyslipidemia, atherosclerosis, ASCVD (i.e., ischemic stroke and MI), and heart failure. The pathological function of elevated furin and its substrates is supported by direct and indirect preclinical data. In clinical practice, treatment of CVDs requires polypharmacy, which leads to poor compliance and potential adverse reactions due to drug interactions. Therefore, targeting furin might be an interesting treatment option, particularly for atherosclerosis and ASCVD, given that it could simultaneously attenuate the activity of several pathogenic substrates. At present, several furin inhibitors, including small molecules^158−161^ and peptides,^101,162−164^ have been developed, but their clinical implications remain inconclusive. Furin inhibitor α-1-PDX recently demonstrated *in vivo* benefits in atherosclerosis,^101^ which supports the therapeutic significance of targeting furin. Given that several PCs show some redundant or complementary function, selective furin inhibitors might have an acceptable safety profile.^48^ For example, PCSK6 has 70% structural homology with furin, and it plays an essential role in blood pressure control via the activation of ANP^48−50^ and BNP^47^ in cardiomyocytes. In addition, other PCSKs, including PCSK6, appear to compensate for pro-insulin receptor processing in the absence of furin in the liver.^68^ Therefore, targeting furin might not dramatically worsen comorbid hypertension and diabetes, although a reduction in furin is associated with the risk of these diseases. Additional studies would provide better understanding for mechanistic insight into the functional roles of furin in CVDs or whether furin inhibitors produce significant side effects by increasing blood pressure and plasma glucose levels.
